# Supplementary material for: Respiratory mucosal immune memory to SARS-CoV-2 after infection and vaccination
Source: Nat Commun. 2023 Oct 26;14:6815. doi: 10.1038/s41467-023-42433-w (PMC10603102; doi:10.1038/s41467-023-42433-w)
Supplement: Supplementary file 1 — Supplementary Information [file 41467_2023_42433_MOESM1_ESM.pdf]

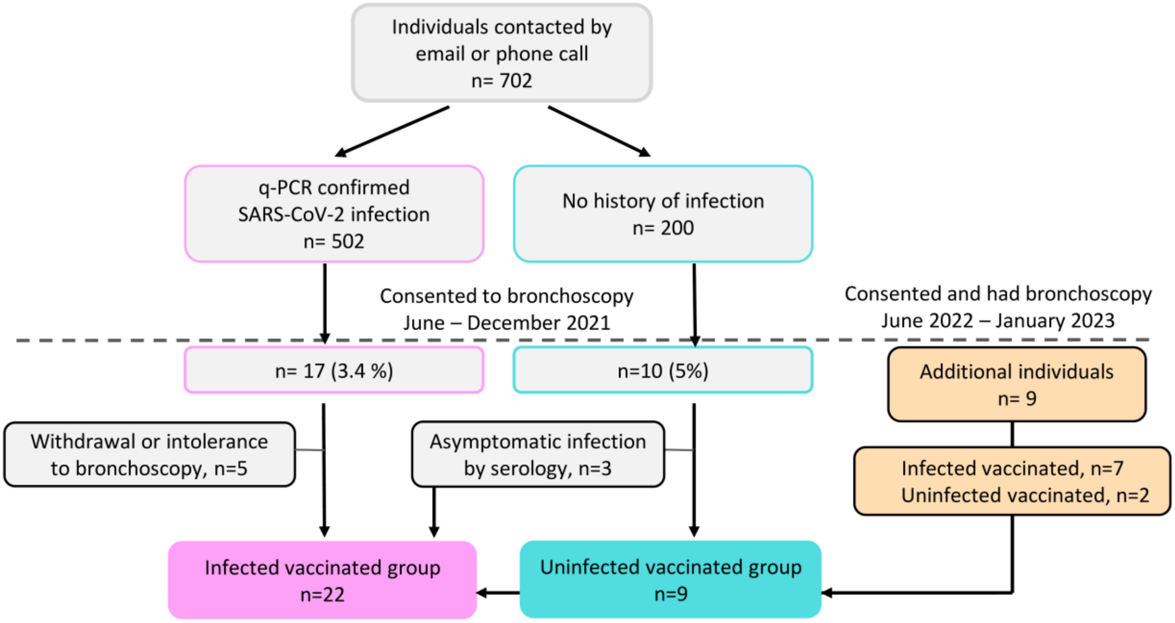

Supplemental Figure 1. Cohort flow chart

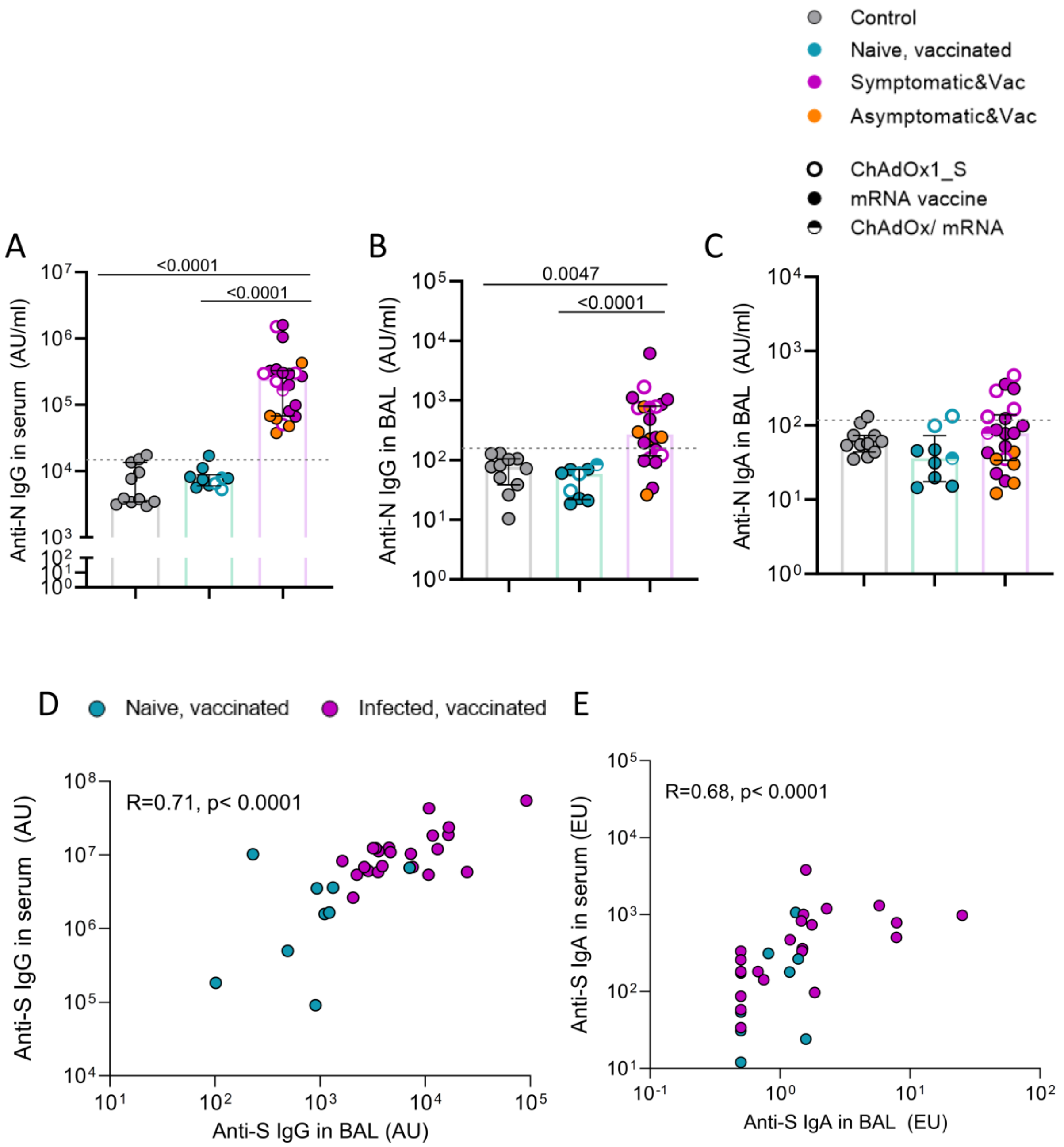

Supplemental Figure 2. Associations between lung mucosa and systemic antibody levels against Spike post SARS-CoV-2 vaccination. (A to C) Levels of anti-N IgG in serum (A) and anti-N IgG (B) and IgA (C) in BAL fluid of control (n=11), uninfected vaccinated (n=9) and infected vaccinated donors (n=22). The limit of assay sensitivity (LOS) per antigen is depicted with dotted black line. Data are presented as median values and interquartile ranges (IQR). Correlation of anti-S IgG (D) and anti-S IgA (E) levels measured in serum and BAL fluid of uninfected vaccinated and infected vaccinated donors. Total n=31 vaccinated individuals. R and p values are shown using two-tailed Spearman rank test. Source data are provided as a Source Data file.

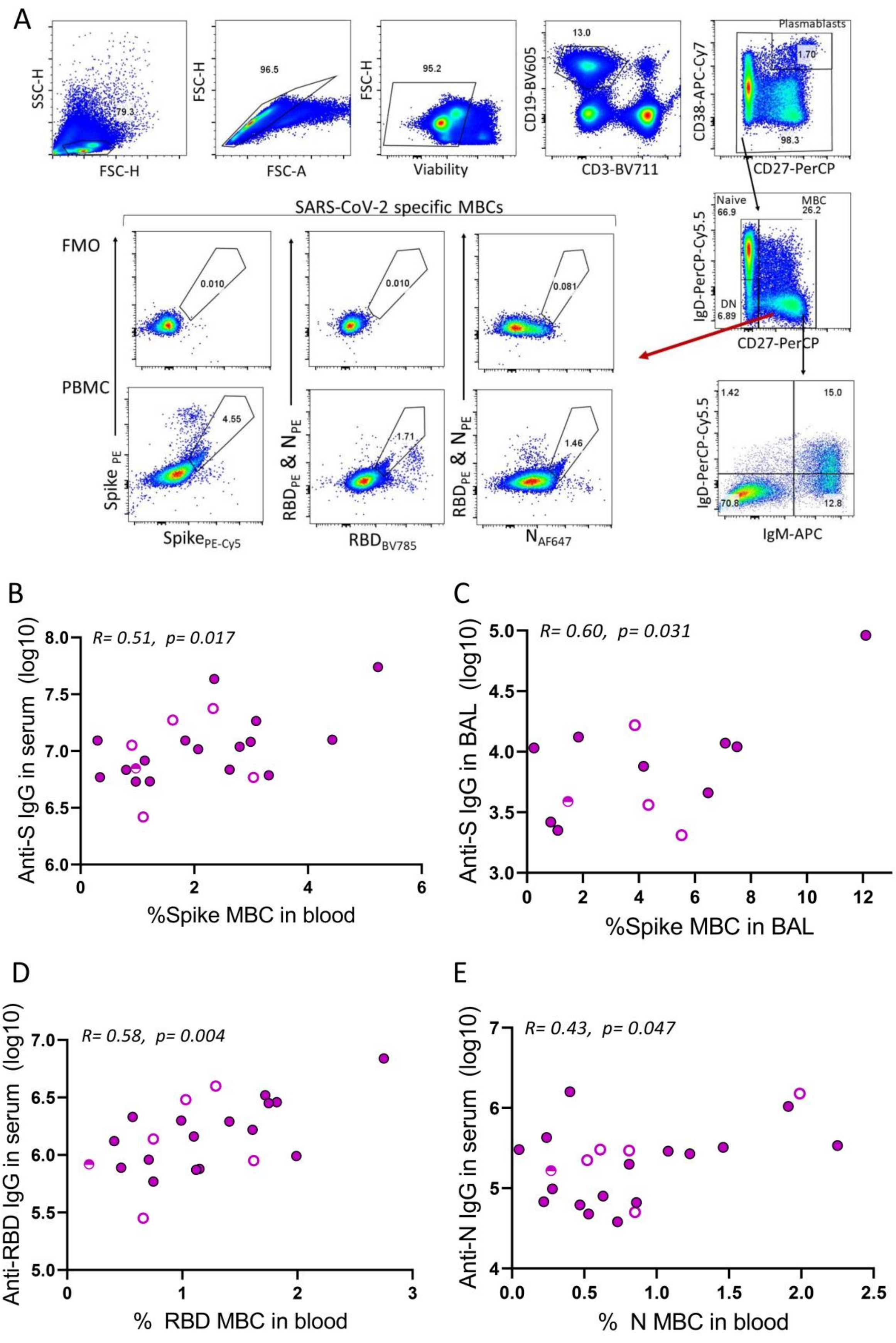
A
SSC-H
FSC-H
79.3
96.5
FSC-A
95.2
Viability
13.0
CD19-BV605
CD3-BV711
Plasmablasts
1.70
CD38-APC-Cy7
98.3
CD27-PerCP
SARS-CoV-2 specific MBCs
FMO
0.010
0.010
0.081
PBMC
4.55
1.71
1.46
Spike PE
Spike PE-Cy5
RBD PE & N PE
RBD BV785
RBD PE & N PE
N AF647
Naive
66.9
MBC
26.2
IgD-PerCP-Cy5.5
DN
6.89
CD27-PerCP
1.42
15.0
70.8
12.8
IgD-PerCP-Cy5.5
IgM-APC
B
R= 0.51, p= 0.017
Anti-S IgG in serum (log10)
%Spike MBC in blood
C
R= 0.60, p= 0.031
Anti-S IgG in BAL (log10)
%Spike MBC in BAL
D
R= 0.58, p= 0.004
Anti-RBD IgG in serum (log10)
% RBD MBC in blood
E
R= 0.43, p= 0.047
Anti-N IgG in serum (log10)
% N MBC in blood

Supplemental Figure 3. Memory B cells and SARS-CoV-2 specific B cells responses in human blood and BAL. A) Gating strategy of B cells in human PBMC or BAL sample, with representative flow cytometry plots of SARS-CoV-2 specific B cells from controls and infected, vaccinated donors. Correlation of the frequency of Spike (B-C) memory B cells detected in blood and BAL, RBD (D) and N (E) measured in the blood with the relevant antibody levels measured in serum or BAL samples in infected vaccinated donors (n=22). Homologous vaccination with ChAdOX1_S or mRNA vaccine is depicted with an open or close circle, respectively and heterologous vaccination with semi-full circle. R and p values are shown using two-tailed Person correlation. Source data are provided as a Source Data file.

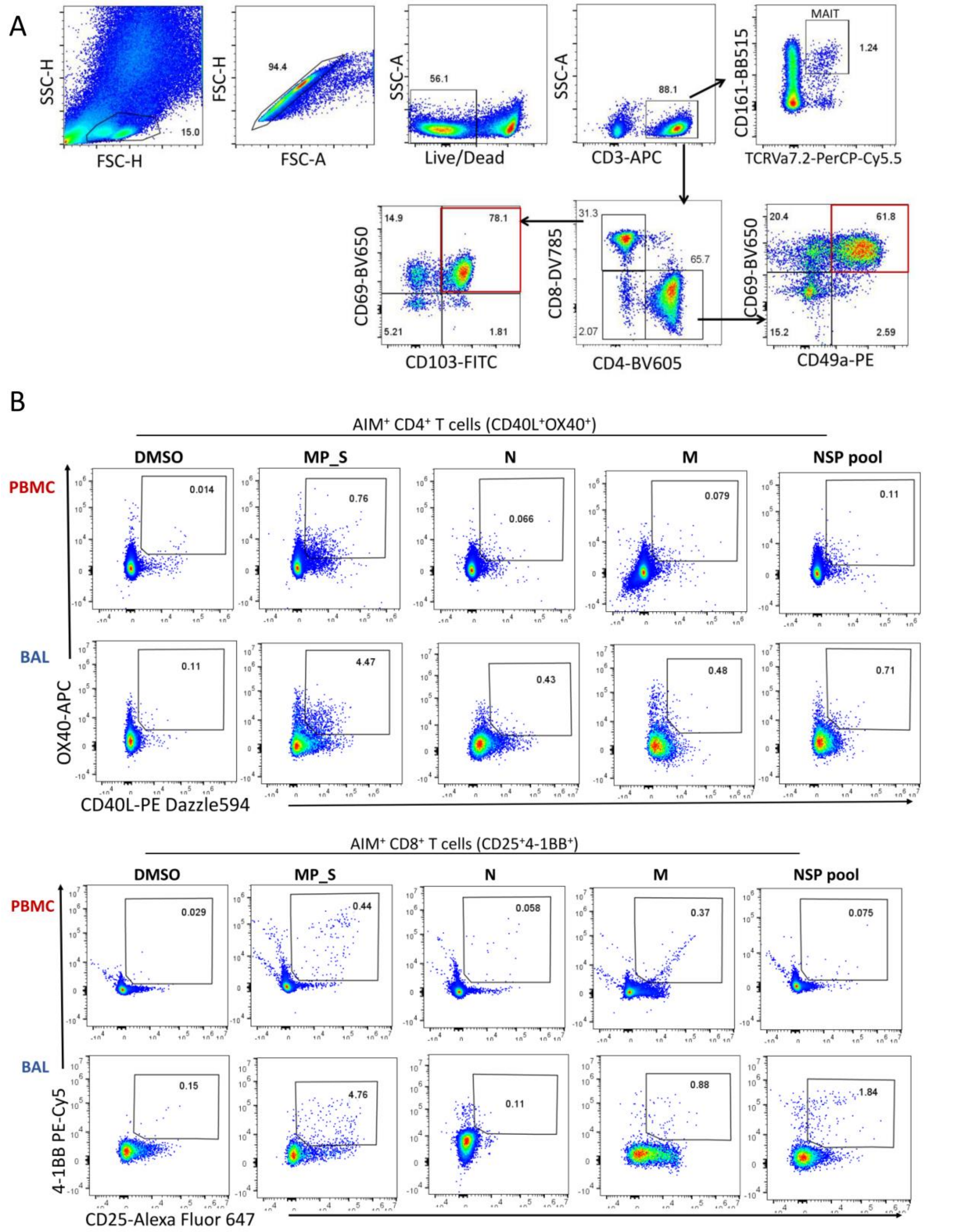

Supplemental Figure 4. Characterisation of T cell populations in blood and bronchoalveolar lavage. A) Example gating of T cell staining after overnight stimulation with SARS-CoV-2 peptide pools. Lymphocytes (SSC-H vs. FSC-H), single cells (FSC-H vs. FSC-A), Live cells (fixable live/dead), CD3$^+$, CD4$^+$ or CD8$^+$ or MAIT cells. Tissue resident memory (TRM) CD4$^+$ and CD8$^+$ T cells were defined as CD4$^+$CD69$^+$CD49a$^+$ and CD8$^+$CD69$^+$CD103$^+$, respectively. B) Representative plots of SARS-CoV-2 specific

CD4⁺ and CD8⁺ T cells in PBMC and BAL of an infected, vaccinated donor after overnight stimulation with SARS-CoV-2 peptide pools. DMSO was used as a negative control.

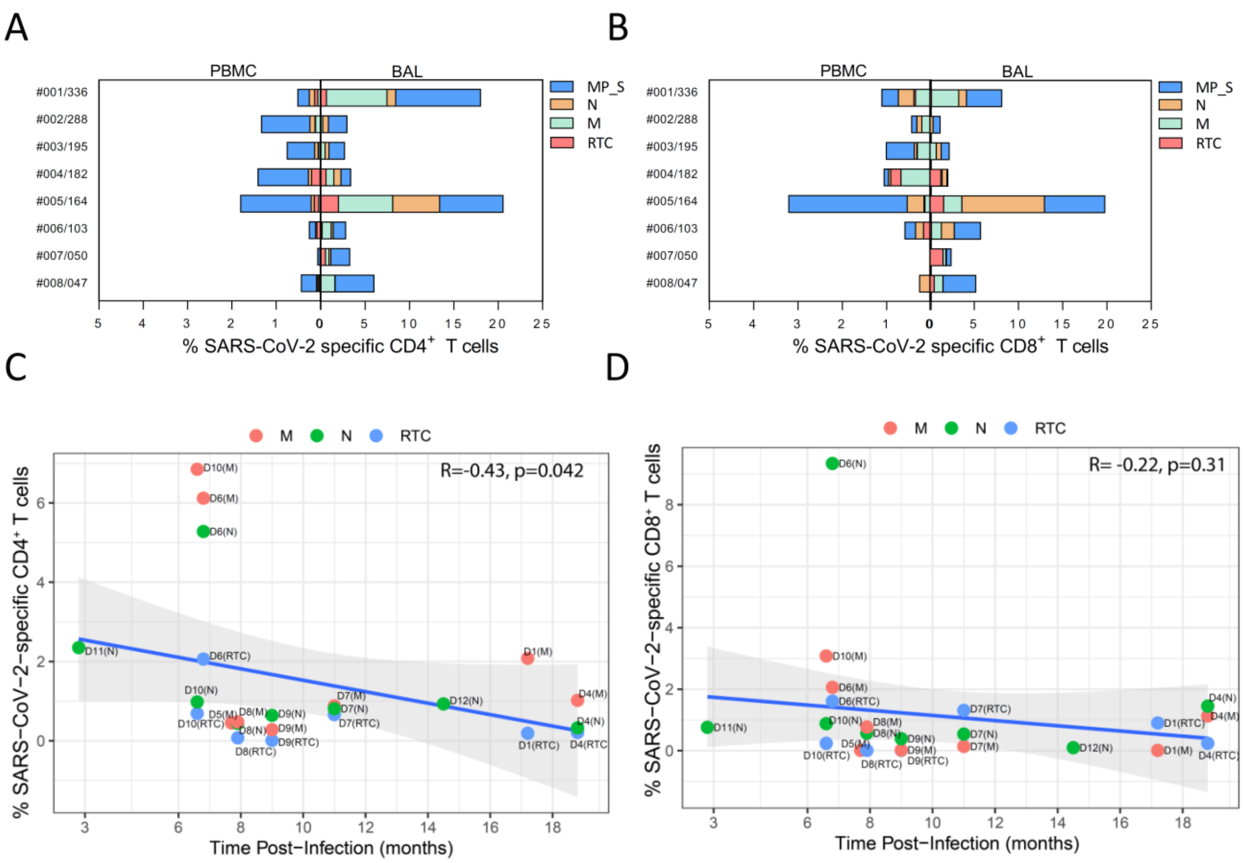

Supplemental Figure 5. T cell specificities hierarchy in blood and lung mucosa and kinetics over time. A-B) Paired analysis of proportions of CD4⁺ and CD8⁺ T cells which recognise SARS-CoV-2 antigens in BAL and paired blood. Statistical differences were determined by two-sided Mann-Whitney U test**.** *(p < 0.05). C-D) Correlation between time post infection from symptoms onset and the frequency of N-, M and RTC- specific CD4⁺ (C) and CD8⁺ T cells (D) detected in BAL of infected vaccinated individuals with symptomatic, PCR-confirmed SARS-CoV-2 infection (n=12). Results of two-tailed Spearman correlation test and linear regression line with 95% confidence interval (grey shading) are shown. Source data are provided as a Source Data file.

Marker	Fluorochrome	Clone	Provider	Reference	Dilution
CD21	SB436	HB5	Thermofisher	62-0219-42	1/33
CD95 (Fas)	BV421	DX2	Biolegend	305624	1/33
CD11c	Pacific Blue	Bu15	Biolegend	337212	1/20
CD185 (CXCR5)	BV480	RF8B2	BD Biosciences	566142	1/33
CD19	BV605	HIB19	Biolegend	302244	1/20
CD20	BV650	2H7	Biolegend	302336	1/20
CD3	BV711	SK7	Biolegend	344838	1/33
CD27	PE-eFluor610	O323	Thermofisher	61-0279-42	1/33
CD24	FITC	ML5	Biolegend	311104	1/33
IgD	PerCPCy5.5	IA6-2	Biolegend	348208	1/33
CCR10	PECy7	13E11	Biolegend	362108	1/20
IgM	APC	SA-DA4	ThermoFisher	17-9998-42	1/33
CD71	AF700	M-A712	BD Biosciences	563769	1/33
CD38	APC-Cy7	HIT2	Biolegend	303534	1/50
Live & Dead	e506	NA	Thermofisher	65-0866-14	1/4

Supplemental Table 1. Summary of antibody cocktail used for B cell immunophenotyping. A multiparametric flow cytometry panel consisted of 14 monoclonal antibodies and a viability dye was used to immunophenotype global and memory B cells in blood and BAL samples from the 3 study groups. Electronic compensation was set using CompBeads (BD Biosciences), Arc beads (ThermoFisher) and using the Spectral Flow automated unmixing software (Aurora, Cytek) according to manufacturer's instructions.

Marker	Fluorochrome	Clone	Provider	Reference	Dilution
CD45RA	BV750	H100	BD Biosciences	747435	1/100
CD197 (CCR7)	BV480	3D12	BD Biosciences	566099	1/20
CD3	APC-Cy7	SK7	Biolegend	344818	1/65
CD4	BV605	SK3	Biolegend	344646	1/33
CD8	BV785	SK1	Biolegend	344740	1/50
CD185 (CXCR5)	SB436	MU5UBEE	Thermofisher	62-9185-42	1/20
CD183 (CXCR3)	BV510	G025H7	Biolegend	353726	1/33
CD196 (CCR6)	PECy7	G034E3	Biolegend	353418	1/33
TCRVa7.2	PerCPCy5.5	3C10	Biolegend	351710	1/33
CD161	BB515	REA631	Miltenyi Biotech	130-122-808	1/100
CD69	BV650	FN50	Biolegend	310934	1/33
CD154 (CD40L)	PE/Dazzle594	24-31	Biolegend	310840	1/100
CD137 (4-1BB)	PECy5	4B4-1	Biolegend	309808	1/33
CD134 (OX40)	APC	ACT35	Biolegend	350008	1/100
CD25	AF647	BC96	Biolegend	302617	1/33
Live & Dead	e506	NA	Thermofisher	65-0866-14	1/4

Supplemental Table 2. Summary of antibody cocktail used for T cell immunophenotyping and identification of antigen-specific T cells in blood using AIM assay**.**

Marker	Fluorochrome	Clone	Provider	Reference	Dilution
CD45	BV750	HI30	BD Biosciences	747321	1/100
CD3	APC-Cy7	SK7	Biolegend	344818	1/65
CD4	BV605	SK3	Biolegend	344646	1/33
CD8	BV785	SK1	Biolegend	344740	1/50
CD183 (CXCR3)	BV510	G025H7	Biolegend	353726	1/33
TCRVa7.2	PerCPCy5.5	3C10	Biolegend	351710	1/33
CD161	BB515	REA631	Miltenyi Biotech	130-122-808	1/100
CD69	BV650	FN50	Biolegend	310934	1/33
CD103	FITC	Ber-ACT8	Biolegend	350204	1/33
CD49a	PE	TS2/7	Biolegend	328304	1/100
CD154 (CD40L)	PE/Dazzle594	24-31	Biolegend	310840	1/100
CD137 (4-1BB)	PECy5	4B4-1	Biolegend	309808	1/33
CD134 (OX40)	APC	ACT35	Biolegend	350008	1/100
CD25	AF647	BC96	Biolegend	302617	1/33
Live & Dead	e506	NA	Thermofisher	65-0866-14	1/4

Supplemental Table 3. Summary of antibody cocktail used for T cell immunophenotyping and identification of antigen-specific T cells in BAL using AIM assay**.**
