# Supplementary material for: Respiratory mucosal immune memory to SARS-CoV-2 after infection and vaccination
Source: Nat Commun. 2023 Oct 26;14:6815. doi: 10.1038/s41467-023-42433-w (PMC10603102; doi:10.1038/s41467-023-42433-w)
Supplement: Supplementary file 3 — Reporting Summary [file 41467_2023_42433_MOESM3_ESM.pdf]

nature portfolio

Corresponding author(s): Elena Mitsi, Daniela Ferreira

Last updated by author(s): 14-September-2023

Reporting Summary

Nature Portfolio wishes to improve the reproducibility of the work that we publish. This form provides structure for consistency and transparency in reporting. For further information on Nature Portfolio policies, see our Editorial Policies and the Editorial Policy Checklist.

Statistics

For all statistical analyses, confirm that the following items are present in the figure legend, table legend, main text, or Methods section.

n/a	Confirmed	
☐	☒	The exact sample size (*n*) for each experimental group/condition, given as a discrete number and unit of measurement
☐	☒	A statement on whether measurements were taken from distinct samples or whether the same sample was measured repeatedly
☐	☒	The statistical test(s) used AND whether they are one- or two-sided<br>*Only common tests should be described solely by name; describe more complex techniques in the Methods section.*
☒	☐	A description of all covariates tested
☐	☒	A description of any assumptions or corrections, such as tests of normality and adjustment for multiple comparisons
☐	☒	A full description of the statistical parameters including central tendency (e.g. means) or other basic estimates (e.g. regression coefficient) AND variation (e.g. standard deviation) or associated estimates of uncertainty (e.g. confidence intervals)
☐	☒	For null hypothesis testing, the test statistic (e.g. *F*, *t*, *r*) with confidence intervals, effect sizes, degrees of freedom and *P* value noted<br>*Give P values as exact values whenever suitable.*
☒	☐	For Bayesian analysis, information on the choice of priors and Markov chain Monte Carlo settings
☒	☐	For hierarchical and complex designs, identification of the appropriate level for tests and full reporting of outcomes
☐	☒	Estimates of effect sizes (e.g. Cohen's *d*, Pearson's *r*), indicating how they were calculated

Our web collection on statistics for biologists contains articles on many of the points above.

Software and code

Policy information about availability of computer code

Data collection	Optical density data were acquired using an Omega microplate reader at 405nm and Gen5 V3.09 software. Flow cytometry data were acquired using an Aurora flow cytometer with manufacturer's software (SpectroFlo v1.1)
Data analysis	Flow cytometry analysis were performed using Flowjo software (V.10). Statistical analysis was done in R software version 4.0.3 or Graphpad Prism version 9.0. R package used was rstatix (version 4.2.3).

For manuscripts utilizing custom algorithms or software that are central to the research but not yet described in published literature, software must be made available to editors and reviewers. We strongly encourage code deposition in a community repository (e.g. GitHub). See the Nature Portfolio guidelines for submitting code & software for further information.

Data

Policy information about availability of data

All manuscripts must include a data availability statement. This statement should provide the following information, where applicable:

- Accession codes, unique identifiers, or web links for publicly available datasets
- A description of any restrictions on data availability
- For clinical datasets or third party data, please ensure that the statement adheres to our policy

All data generated or analysed during this study are included in this published article (and its supplementary information files). A Source Data file is provided with this paper. All relevant data are also available from the authors.

April 2023

Research involving human participants, their data, or biological material

Policy information about studies with human participants or human data. See also policy information about sex, gender (identity/presentation), and sexual orientation and race, ethnicity and racism.

Reporting on sex and gender	Detailed information is provided in Table 1 and is reported as female % per study group.
Reporting on race, ethnicity, or other socially relevant groupings	The study was perfomed in Merseyside county (North-West of England), hence the majority of study participants were British or of any other white background.
Population characteristics	Population characteristics, SARS-CoV-2 vaccination status and history of infection have been described in Methods and in Table 1.<br>The study included a cohort of SARS-CoV-2 vaccinated individuals (n=31). A subset of them had experienced PCR-confirmed symptomatic infection (n=17) or serologically confirmed asymptomatic infection (n=5), whereas the remaining vaccinees had remained infection-vaccine (uninfected vaccinated, n=9).<br>Also, 11 unexposed, non-vaccinated donors were used as healthy pre-pandemic controls.
Recruitment	All volunteers gave written informed consent and were invited to participate into the study (Umbrella Bronchscopy study) via email and text messages. Selection bias may have been introduced, as the majority of study participants were recruited from the county of Merseyside and therefore they were individuals from Caucasians descend. This did not allow us to assess the role of genetics and enviromental influences neither to expand study's conclusions to other geographical regions.
Ethics oversight	Ethical approval was given by the NorthWest National Health Service Research Ethics Committee (REC no. 18/NW/0481 and Human Tissue Authority licensing number: 12548)

Note that full information on the approval of the study protocol must also be provided in the manuscript.

Field-specific reporting

Please select the one below that is the best fit for your research. If you are not sure, read the appropriate sections before making your selection.

☒ Life sciences ☐ Behavioural & social sciences ☐ Ecological, evolutionary & environmental sciences

For a reference copy of the document with all sections, see nature.com/documents/nr-reporting-summary-flat.pdf

Life sciences study design

All studies must disclose on these points even when the disclosure is negative.

Sample size	No sample size calculation was performed given the exploratory nature of the study. The sample size of bronchoalveolar lavages was determined by logical restrictions, such as clinical staff availability, pre-booked clinical space for bronchoscopies and the availability of samples from uninfected vaccinated individuals.<br>Sample size is reported for each figure and individual dots are shown throughout the paper. Sample size can vary across figure panels depending on which cell type was analysed or stimulations performed, due to BAL sample limited cell recovery.
Data exclusions	No participants or individual samples were excluded from analysis.
Replication	Experiments were not replicated due to human samples limited availability.
Randomization	Samples were allocated into 3 groups based on the donor sinfection and vaccination status, as following:<br>- infection naive, SARS-CoV-2 vaccinated<br>- infected (qPCR and/or serologically confirmed) and SARS-CoV-2 vaccinated<br>- unexposed, unvaccinated healthy controls (pre-pandemic samples)
Blinding	Operators were blinded to group allocation during the experiments.

Reporting for specific materials, systems and methods

We require information from authors about some types of materials, experimental systems and methods used in many studies. Here, indicate whether each material, system or method listed is relevant to your study. If you are not sure if a list item applies to your research, read the appropriate section before selecting a response.

Materials & experimental systems

n/a	Involved in the study
[ ]	[x] Antibodies
[x]	[ ] Eukaryotic cell lines
[x]	[ ] Palaeontology and archaeology
[x]	[ ] Animals and other organisms
[ ]	[x] Clinical data
[x]	[ ] Dual use research of concern
[x]	[ ] Plants

Methods

n/a	Involved in the study
[x]	[ ] ChIP-seq
[ ]	[x] Flow cytometry
[x]	[ ] MRI-based neuroimaging

Antibodies

Antibodies used

Flow cytometry antibodies

B cell immunophenotyping panel
Marker Fluorochrome Clone Provider Reference Dilution
CD45RA BV750 H100 BD Biosciences 747435 (1/100)
CD197 (CCR7) BV480 3D12 BD Biosciences 566099 (1/20)
CD3 APC-Cy7 SK7 Biolegend 344818 (1/65)
CD4 BV605 SK3 Biolegend 344646 (1/33)
CD8 BV785 SK1 Biolegend 344740 (1/50)
CD185 (CXCR5) SB436 MU5UBEE Thermofisher 62-9185-42 (1/20)
CD183 (CXCR3) BV510 G025H7 Biolegend 353726 (1/33)
CD196 (CCR6) PECy7 G034E3 Biolegend 353418 (1/33)
TCRVa7.2 PerCPCy5.5 3C10 Biolegend 351710 (1/33)
CD161 BB515 REA631 Miltenyi Biotech 130-122-808 (1/100)
CD69 BV650 FN50 Biolegend 310934 (1/33)
CD154 (CD40L) PE/Dazzle594 24-31 Biolegend 310840 (1/100)
CD137 (4-1BB) PECy5 4B4-1 Biolegend 309808 (1/33)
CD134 (OX40) APC ACT35 Biolegend 350008 (1/100)
CD25 AF647 BC96 Biolegend 302617 (1/33)
Live & Dead e506 NA Thermofisher 65-0866-14 (1/4)

T cell blood and BAL panels:
Marker Fluorochrome Clone Provider Reference Dilution
CD45RA BV750 H100 BD Biosciences 747435 (1/100)
CD197 (CCR7) BV480 3D12 BD Biosciences 566099 (1/20)
CD3 APC-Cy7 SK7 Biolegend 344818 (1/65)
CD4 BV605 SK3 Biolegend 344646 (1/33)
CD8 BV785 SK1 Biolegend 344740 (1/50)
CD185 (CXCR5) SB436 MU5UBEE Thermofisher 62-9185-42 (1/20)
CD183 (CXCR3) BV510 G025H7 Biolegend 353726 (1/33)
CD196 (CCR6) PECy7 G034E3 Biolegend 353418 1(1/33)
TCRVa7.2 PerCPCy5.5 3C10 Biolegend 351710 (1/33)
CD161 BB515 REA631 Miltenyi Biotech 130-122-808 (1/100)
CD69 BV650 FN50 Biolegend 310934 1/33
CD154 (CD40L) PE/Dazzle594 24-31 Biolegend 310840 (1/100)
CD137 (4-1BB) PECy5 4B4-1 Biolegend 309808 (1/33)
CD134 (OX40) APC ACT35 Biolegend 350008 (1/100)
CD25 AF647 BC96 Biolegend 302617 (1/33)
CD103 FITC Ber-ACT8 Biolegend 350204 (1/33)
CD49a PE TS2/7 Biolegend 328304 (1/100)
Live & Dead e506 NA Thermofisher 65-0866-14 1/4

Validation

All antibodies were purchased from well established manufactures and were validated by the vendor for species and target. e.g. BD biosciences, Biolegend in knock-out primary model systems to ensure biological accuracy in ISO 9001 certified facilities.
Side-by-side lot comparisons were performed. Details of antibody clones have been included for cross-referencing of manufacturing company specification/validation processes. We further validated antibodies by titration to optimal concentrations and by using positive controls, where possible. Fluorencence minus one staining and unstimulated wells were used to define gates in Flowjo for all FACS assays. Negative controls were included in each run and positive controls where possible. All data are presented as background subtracted as described in the methods. The following statements were made by the manufacturer's:

BD: validated for flow cytometry (routinely tested) and QC tested
Biolegend: Each lot product is validated by QC testing with a series of titration dilutions by Biolegend

Thermofisher: CD185 (CXCR5) SB436 MU5UBEE- This MU5UBEE antibody has been pre-diluted and tested by flow cytometric analysis of normal human peripheral blood cells.
Miltenyi Biotech: In order to compare the epitope specificity of an antibody, the clone being used is compared with other known clones recognizing the same antigen in a competition assay.Based on the fluorescence signal obtained, the clones were identified as recognizing completely overlapping (++), partially overlapping (+), or completely different epitopes (-) of the marker.

Clinical data

Policy information about clinical studies
All manuscripts should comply with the ICMJEguidelines for publication of clinical research and a completedCONSORT checklist must be included with all submissions.

Clinical trial registration: 18/NW/0481

Study protocol: The study protocol is available upon request from authors

Data collection: The study was performed in Liverpool School of Tropical Medicine, UK. Clinical data were collected using routine diagnostics, recorded on Clinical Research Forms (CRF) and entered to RedCap platform.

Outcomes:
Primary outcome:
To investigate differential immune responses in the lung of healthy participants compared to patient and/or vaccinated groups.

Secondary Outcomes:
To assess bacterial immune responses in the lung in healthy participants and different patient or vaccinated groups.
To assess levels of inflammation in the respiratory mucosa
To evaluate the upper and lower airway microbiome in healthy participants and different patient groups.

Flow Cytometry

Plots

Confirm that:

- [x] The axis labels state the marker and fluorochrome used (e.g. CD4-FITC).
- [x] The axis scales are clearly visible. Include numbers along axes only for bottom left plot of group (a 'group' is an analysis of identical markers).
- [x] All plots are contour plots with outliers or pseudocolor plots.
- [x] A numerical value for number of cells or percentage (with statistics) is provided.

Methodology

Sample preparation: Information about sample preparation is provided in the Methods.
All FACS was performed on cryopreserved samples. PBMC were isolated from heparinised blood samples by density gradient separation, layered on Ficoll-Paque in SepMate tubes. BAL cells were separated from the supernatant by centrifugation. Isolated PBMC and BAL cells were cryopreserved in CTL-CryoABC media kit (Immunospot).
On the day of sample analysis, both PBMC and BAL samples were thawed. For BAL samples, non-adherent cells were separated from alveolar macrophages using an adherence step.

Instrument: Aurora cytometer (Cytek Biosciences)

Software: Flowjo V.10

Cell population abundance: For BAL samples, non-adherent cells were stimulated after 3 hour resting at 37oC, 5% CO2. PBMCs were stimulated after 2 hours resting t 37oC, 5% CO2. Both PBMC and BAL cells were stained and run without sorting or enrichment.

Gating strategy: Example gating strategy and plots are give in Extended Data Figure 2 (B cells in PBMC and BAL) and Extended Data Figure 3 (T cells in PBMC and BAL).

- [x] Tick this box to confirm that a figure exemplifying the gating strategy is provided in the Supplementary Information.
